# Real-Life Performance of a Commercially Available AI Tool for Post-Traumatic Intracranial Hemorrhage Detection on CT Scans: A Supportive Tool

**DOI:** 10.3390/jcm14134403

**Published:** 2025-06-20

**Authors:** Léo Mabit, Maryne Lepoittevin, Martin Valls, Clément Thomas, Rémy Guillevin, Guillaume Herpe

**Affiliations:** 1 Radiology Department, University Hospital of Poitiers, 2 rue de la Milétrie, 86000 Poitiers, France; leo.mabit@chu-poitiers.fr (L.M.);; 2 Institut National de la Santé et de la Recherche Médicale U1313—Ischémie-Reperfusion, Metabolisme et Inflammation Sterile en Transplantation, University Hospital of Poitiers, 2 rue de la Milétrie, 86021 Poitiers, Cedex 9, France; 3 Data Analysis and Computations Through Imaging Modeling-Mathématiques, Imagerie, Santé—Laboratoire de Mathématiques et Applications, 86073 Poitiers, Cedex 9, France

**Keywords:** intracranial hemorrhage, traumatic brain injury, artificial intelligence, brain CT scan, emergency radiology

## Abstract

**Background**: Traumatic brain injury (TBI) is a major cause of morbimortality in the world, and it can cause potential intracranial hemorrhage (ICH), a life-threatening condition that requires rapid diagnosis with computed tomography (CT). Artificial intelligence tools for ICH detection are now commercially available. **Objectives**: Investigate the real-world performance of qER.ai, an artificial intelligence-based CT hemorrhage detection tool, in a post-traumatic population. **Methods**: Retrospective monocentric observational study of a dataset of consecutively acquired head CT scans at the emergency radiology unit to explore brain trauma. AI performance was compared to ground truth determined by expert consensus. A subset of night shift cases with the radiological report of a junior resident was compared to the AI results and ground truth. **Results**: A total of 682 head CT scans were analyzed. AI demonstrated a sensitivity of 88.8% and a specificity of 92.1% overall, with a positive predictive value of 65.4% and a negative predictive value of 98%. AI’s performance was comparable to that of junior residents in detecting ICH, with the latter showing a sensitivity of 85.7% and a high specificity of 99.3%. Interestingly, the AI detected two out of three ICH cases missed by the junior residents. When AI assistance was integrated, the combined sensitivity improved to 95.2%, and the overall accuracy reached 98.8%. **Conclusions**: This study shows better performance from AI and radiologist residents working together than each one alone. These results are encouraging for rethinking the radiological workflow and the future of triage of this large population of brain traumatized patients in the emergency unit.

## 1. Introduction

Traumatic brain injury (TBI) is a major health concern, with an estimated 69 million individuals suffering from TBI from all causes each year [1,2]. Potentially associated intracranial hemorrhage (ICH) is a life-threatening condition with substantial mortality, morbidity, and reduced quality of life [3,4,5,6].

Therefore, identifying even minor hemorrhages promptly is mandatory to ensure that patients receive the appropriate treatment without delay. Early detection is beneficial not only for patients with ICH but also for those without it who can be discharged according to the local clinical protocol. This approach helps with the optimal use of the healthcare system’s costly trauma resources.

Non-contrast CT scans are the most widely used imaging modality to diagnose acute ICH due to various advantages over the more precise though expensive and limitedly available MRI [7].

Acute intracranial hemorrhage (AIH) cases are common at major Level 2 trauma centers and above, which might not always be best equipped to offer 24/7 neurological care. At nighttime, senior neuroradiologists might not be present, and in academic medical centers, the organization relies on radiology residents to provide after-hours coverage, with the attending radiologist reviewing the following day or providing teleradiology solutions [8,9,10]. Radiological workload has drastically increase over the last 20 years, especially during night shifts, where reporting might be delayed and mistakes made [11,12,13,14,15,16,17,18].

Technological innovations like AI (artificial intelligence) solutions can be a great assistance for reporting radiologists and can help in detecting complex, small, and difficult-to-spot hemorrhages on a CT. AI specifically trained to detect, localize, and estimate AIH has demonstrated good results in studies. Some algorithms can even classify subtypes of AIH, thus helping clinicians with appropriate management of patients [19,20].

Many AI algorithms are commercially available in France for ICH detection [21,22]. However, not much data exists on the real-world performance of AI, especially in the local French population from trauma centers.

The main aim of our study is to determine the performances of AI software in detecting AIH, especially during nighttime, when junior physicians are reading CTs and making preliminary diagnoses. The study also aimed to identify whether AI can be used as an assistance tool for junior radiologists to feel more confident in their reporting so that they can use the AI appropriately.

## 2. Materials and Methods

This study was reviewed and approved by the Institutional Review Board of CERIM (CRM-2401-400). Data processing steps were compliant with the European General Data Protection Regulation (GDPR). Patients consent was waived by the IRB.

### 2.1. Study Design

This retrospective, consecutive real-world evaluation study evaluated an AI diagnostic-aided brain hemorrhage tool for use by emergency radiologists. An external dataset of consecutive patients referred to our tertiary trauma center (University Hospital Center, Poitiers, France) for brain trauma. Brain CT scans were interpretated with or without AI assistance depending on the period of the study.

The interpretation performance in these two read-out sessions was evaluated using expert ground truth as reference. The AI algorithm included features for detection of intracranial hemorrhage, midline shift, and bone fracture, but our study focused on ICH.

#### 2.1.1. Data Collection

Participant data was collected consecutively from one institution. Data analysis was centralized and evaluated with the same visualization tool.

#### 2.1.2. Data Selection

Images of brain CT scans performed consecutively between 21 August 2023 and 21 January 2024 in our emergency radiology unit on 2 different CT scanners (Go Top, Siemens, Erlangen, Germany; Aquilion One, Toshiba, Tokyo, Japan) were collected.

A subset of this database was selected to build an external validation dataset of traumatic patients according to the following criteria: mild to severe brain trauma; patients over the age of 18 y.o.

According to clinical condition, patients with a Glasgow Coma Scale ≥ 13 in the emergency department’s initial evaluation were considered to have mild or minor brain trauma. Severe or major trauma was considered when the Glasgow Coma Scale was <13 in the emergency department’s initial evaluation or when a full-body CT scan was carried out in a polytraumatic context.

Patients under the age of 18 at the time of image acquisition and brain CT scans for which AI algorithm results were not available in our PACS station were excluded from the study.

**Figure 1:**
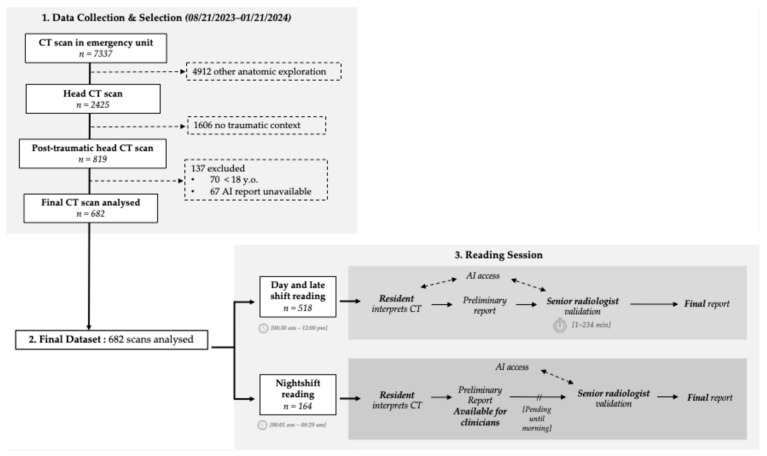
Study design and flowchart.

Mechanisms of trauma were collected for the subanalysis of the night shift subset. The study workflow is illustrated in Figure 1, and the final dataset demographics are described in Table 1.

### 2.2. Intervention

#### 2.2.1. Readers

The readers in the study were composed of 28 senior radiologists with over five years of experience in emergency radiology and 17 in-training residents. A list of the readers along with their years of experience is provided within the Supplementary Material.

#### 2.2.2. Reading Workflow

During the study period, the assigned task for readers involved interpreting brain computed tomography scans and documenting the presence of any intracranial hemorrhages, including the location of the hemorrhage if identified. Initial evaluations were conducted by a resident who produced a preliminary report. This report was then subjected to subsequent validation by a senior radiologist, with mean validation times ranging from 1 to 234 min.

Throughout the day shift and the late shift (08:30 a.m. to 12:00 p.m.), the resident and the senior had instantaneous access to the AI results, and a final report was issued immediately.

Throughout the night shifts, defined as the time from 00:01 a.m. to 08:29 a.m., residents did not have access to artificial intelligence algorithm outcomes when preparing their preliminary reports. The integration of AI algorithm results into the final reports occurred the next morning, when a senior radiologist performed the validation. The study workflow is shown in Figure 1.

#### 2.2.3. Reading Setup

Readers used the same reading setup as the expert, which consisted of a PACS workstation (Change Healthcare Radiology Station v14, Maincare, Vancouver, BC, Canada). During the AI-assisted reading session, if anomalies were detected, the AI results were presented as a segmented series pinpointing the irregularities, along with a PDF report in the form of a secondary capture. In cases where no anomalies were found, a PDF report indicating “no findings” was displayed. An example of a PDF report is provided in the Supplementary Material.

#### 2.2.4. Ground Truth

Ground truth for the presence or absence of hemorrhage was iteratively determined by a review of every scan and existing radiological reports by a radiologist with 4 years of specialty experience and an expert neuroradiologist with 14 years of experience. They were asked to manually classify whether a brain hemorrhage was present or not. The radiologists had access to all DICOM series and were blinded to the AI results. Wherever there was a discrepancy, a consensus was reached by discussion.

The definitions used to define intracranial hemorrhage were as follows: any type of bleeding within the cranial vault, including the brain parenchyma and meningeal space. For each type of hemorrhage, the software detects and localizes the following bleeds: epidural hemorrhage, subdural hemorrhage, subarachnoid hemorrhage, intraparenchymal hemorrhage, and intraventricular hemorrhage.

### 2.3. AI Model

The qER-NCCTv1.0 software (Qure.ai, Mumbai, India) was used in our study. The qER-NCCTv1.0 device consists of a set of pre-trained segmentation convolutional neural networks (CNNs) that are deployed in parallel to provide pixel-wise segmentation output for the target structures. The software contains four individual algorithms, each of which detects one of the four abnormalities—intracranial bleeds, cranial fractures, mass effect, or midline shift. A set of pre-trained deep learning models are applied to the incoming images to detect the target abnormalities. The core component of each algorithm is a classification convolutional neural network (CNN) that has been trained to detect a specific abnormality from head CT scan images. This core component is coupled with a pre-processing module that transforms the CT scan series to a set of images and a post-processing module that combines slice-level output probabilities to a scan-level triage result for each abnormality.

### 2.4. Statistical Analysis

Performance evaluation consisted of calculation of the sensitivity (% of correct predictions on pathological cases), specificity (% of correct predictions on healthy cases), positive and negative predictive values, and accuracy (% of correct predictions on all cases). As local protocols require specialized neurosurgical advice for any intracranial hemorrhage, our study only focused on ICH detection performance to explore the triage capacity of such a tool, and AI subclassification of ICH has not been studied.

Calculation was performed for AI alone versus ground truth for the whole dataset and for junior radiologist versus ground truth for the night shift subset. Combined metrics of AI and junior radiologists were obtained by crossing their individual performances.

Statistical analysis was performed using R software (version 12.0, R Foundation for Statistical Computing, Vienna, Austria). Statistical tests were considered significant at a threshold of *p* < 0.05. 

### 2.5. Challenging Cases

All instances of false negatives identified by artificial intelligence were subsequently examined by a neuroradiologist and a neurosurgeon. Their review focused on assessing the severity and potential clinical consequences of each case, employing the NIRIS scoring system (NeuroImaging Radiological Interpretation System) for evaluation [23]. Additionally, all false positives were visually re-reviewed to determine potential reasons for failure of the model. 

## 3. Results

During the data collection time, a total of 7337 CT scans were performed, 2425 of which were for cerebral exploration. Among these, 819 were in a post-traumatic context, representing 33.8% of the total number of head CT scans and 11.2% of all CT scans performed in our emergency radiology unit during this period. A total of 137 CT scans met the exclusion criteria due to the lack of an available AI report in the PACS station (49%, 67/137) and due to age < 18 y.o. (51%, 70/137). A final dataset of 682 CT scans was analyzed, among which 164 were during the night shift. According to ground truthing, a total of 98 ICHs occurred in the study, with a prevalence of 14.4%. The highest proportion of ICH was found in severe trauma (38.4%). A total of 21 ICHs occurred in the night shift subset (12.8%, 21/164). Three discrepancies were registered between radiologist reports and ground truthing, only in the day shift session. The characteristics of ICH are shown in Table 2. 

### 3.1. Overall AI Performance

For all scans, AI detected hemorrhage with a sensitivity of 88.8% (95% CI: 81–93.6%) and a specificity of 92.1% (95% CI: 89.7–94%). The positive predictive value was 65.4% (95% CI: 57–73%). The negative predictive value reached 98% (95% CI: 96.5–98.9%). The AUC was 0.9 and the overall accuracy was recorded as 91.6%. 

When analyzing the results by trauma severity, the AI showed a sensitivity of 85.5% (95% CI: 73.8–92.4%) and a specificity of 92.8% (95% CI: 90.3–94.7%) for minor traumas. For major traumas, the sensitivity was at 93% (95% CI: 81.4–97.6%).

Details of the AI performance are provided in Table 3.

### 3.2. Night Reads Subset

During the night shift, regarding the mechanism of the trauma, low-intensity fall constituted the majority (70%, 115/164), followed by public road accident (19%, 31/164), direct trauma (6%, 10/164), and high-intensity fall (5%, 8/164).

The qER.ai intrinsic and extrinsic performance were similar in this subset and in the general set, with a sensitivity of 90.5% (95% CI: 71.1–97.4%) and a specificity of 95.8% (95% CI: 91.2–98.1%). The positive predictive value was 76% (95% CI: 56.6–88.6%), with a negative predictive value of 98.6% (95% CI: 94.9–99.6%). The accuracy stood at 95.2%.

Junior residents’ sensitivity was 85.7% (95% CI: 65.4–95%), while their specificity was 99.3% (95% CI: 96.2–99.9%). The positive predictive value was 94.7% (95% CI: 75.4–99.1%) and the negative predictive value was 98% (95% CI: 94.1–99.3%), with an accuracy of 97.6%.

Of the three ICHs missed by the juniors, AI detected two of them. When combining AI and junior radiologists, the sensitivity was 95.2% (95% CI: 77.3–99.2%), the specificity was 99.3% (95% CI: 96.2–99.9%), the positive predictive value was 95.2% (95% CI: 77.3–99.2%), the negative predictive value was 99.3% (95% CI: 96.2–99.9%), and the overall accuracy was 98.8%. Details of the AI and junior performance are provided in Table 4.

### 3.3. Challenging Cases

Three differences between the final report and the expert ground truth were noted. The discrepancies included one instance where intracranial hemorrhage (ICH) was missed, as the CT was erroneously reported as normal, and two instances where no pathological findings were present yet the report erroneously suggested ICH. The AI solution accurately identified all three of these findings.

The algorithm failed to identify hemorrhage (false negative cases) in 11 cases. Among them, 10 were classified as NIRIS 1, and only one was NIRIS 2 due to minimal intraventricular isolated hemorrhage (Supplementary Data Figure S2). This 83 y.o. patient was admitted in the ER secondary to a minor trauma after a low-intensity fall (GCS 15 at the emergency room) and stayed under surveillance in the neurosurgery unit 4 days before hospital discharge, with no intervention performed. Two patients died during their hospital stay (a 95 y.o. woman who presented a subarachnoid hemorrhage classified as NIRIS 1, who died of a massive ischemic stroke 20 days after admission, and a 101 y.o. man who presented a subarachnoid hemorrhage classified as NIRIS 1, who died of a pulmonary complication of COVID-19). Further details are shown in Table 5. 

Similarly, there were some false positive cases (n = 46), accounted for mainly by the presence of intracranial calcification (16/46, 35%), a hardening beam (9/46, 19%), tumor (7/46, 14%), normal findings such as venous sinuses (6/46, 12%), or others (8/46, 17%).

## 4. Discussion

In this study, we show robust performances of AI detection of ICH in a brain traumatized population with an overall accuracy of 91.6% and a negative predictive value of 98.0%. AI performance to assess the absence of brain hemorrhage is consistent in the overall dataset as well as in the night shift subset. AI has better sensitivity than junior radiologists alone during the night shift, with similar negative predictive value and more false positive findings. When AI and junior radiologists are combined, the accuracy rises to 98.8%. 

An interesting observation in our study is the high rate of false positives and the rare occurrence of false negatives, which, although low, remains critical in emergency settings. Potential improvements to the AI software may help reduce misclassifications and further optimize AI performance. These include improving the quality, quantity, and diversity of the training dataset, particularly by incorporating additional borderline or atypical hemorrhage cases to increase model robustness. Adjusting the loss function to more heavily penalize false negatives may enhance sensitivity, which is critical in emergency contexts. Moreover, enhancing the image preprocessing pipeline—such as through noise reduction, intensity standardization, and artifact suppression—as well as adjusting threshold settings could help minimize both false positives and false negatives. Nevertheless, we acknowledge that we do not have access to the full technical specifications or training details of the AI tool used in our study, which limits our ability to suggest precise algorithmic modifications. However, the proposed strategies reflect commonly adopted and promising directions to improve AI robustness and clinical safety.

Several studies have shown the performance of the AI ICH detection tool [24,25,26], but not in a post-traumatic real-life population nor in a French population. A strong point of our demographic is that every patient treated in the radiology unit for brain trauma is included in our study. Our institution is a major trauma center that caters to a large population and is representative of brain traumatized patients. A multicentric approach was not considered following the unavailability of this commercialized AI tool and the lower capacity of our partners to include the targeted population.

The original design of our study allowed us to highlight the complementarity of the junior radiologists and AI. By increasing the sensitivity, AI has the potential to be an efficient real-time second reader acting here as a safety net [27]. Considering the shortage of radiologists, these findings could imply that it is possible to postpone the senior review of brain CT scans in patients with head trauma. By partnering residents with AI, we could better allocate the senior radiologists’ time, placing CT scan reviews lower on the triage list and giving precedence to the review of more critical cases.

Indeed, it would have been interesting to integrate AI into the workflow to prioritize the reporting of radiological exams with positive AI findings. This has been studied recently in cases of suspicion of ICH and pulmonary embolism, and has shown benefits in wait time (defined as the interval between end-of-examination time and initial report creation time) and turnaround time (the interval between end-of-examination time and report availability) [28,29]. In our real-life study, qER achieves very good prioritization for review, with only one clinically relevant missed case.

Another management scenario to investigate in emergency departments would be assessing the potential clinical impact of directly communicating AI-generated results to emergency clinicians, using them as a flagging system to identify at-risk patients. This could help to save time for triage and improve the care of urgent cases. As this flagging system has already proven its efficiency in stroke patients, one could imagine adapting it to trauma patients [30]. A recent study goes further by assessing the indication of neurosurgery with an AI imaging tool on CTs. It shows an accuracy of 84% and an AUC of 0.89. Such a tool would respond to the need for efficient and fast referrals from primary care centers to tertiary trauma centers with specialized referring clinicians, while avoiding the risks of delayed or unnecessary transfer [31,32]. 

Finally, as AI performance in ICH detection has proven to be efficient, the future utility of AI in the TBI population will probably be to assess white matter integrity and/or to identify post-traumatic non-hemorrhagic lesions [33]. This diagnostic and prognostic empowerment might be a game changer from the next generations of AI.

With the development of AI in the imaging field, the radiological workflow will be redesigned to gain efficiency, improve time to diagnosis, boost productivity, and, hopefully, increase clinical benefits. Two main benefits are expected from AI: radiologist performance empowerment and, maybe most importantly, patient workflow improvement. Several solutions currently offer triage and prioritization options within the radiologist’s workflow. Utilizing such a tool presents three key challenges. The first challenge is the tool’s performance in real-world settings on datasets that accurately reflect the patient population of the facility. Based on experience, although the AI tool performs well with the general population, its effectiveness is diminished for neurosurgical patients with implanted surgical devices (like ventricular shunts, pressure probes, etc.), primarily due to metal-related artifacts. The second challenge involves cultivating the willingness to adopt and utilize AI tools among both radiologists and clinicians. As previously published, radiologists’ and clinicians’ fear of AI inputs might remain a challenge. To achieve effectiveness, triaging tools need to be deeply integrated within the workflows [34,35]. The third challenge is that the integration of IT systems within electronic patient files and clinician information systems presents a significant technical challenge [36]. Given that these solutions might not all be marketed by the same vendors, economic considerations could affect the rollout of AI triaging tools, potentially prolonging the time needed to reconcile efficacy with effectiveness.

In our study, senior radiologists’ reports and ground truth are very similar. Blinding night shift reading allowed us to alleviate automation bias. It therefore would have been interesting to assess the AI consultation report of each radiologist during the daytime session. This also would have been a potential confounder if radiologists’ daytime performance was studied. In everyday life, AI–human interaction is still uncertain and remains part of the explicability of AI that still needs to be addressed. The rate of emerging AI solutions that are effectively used in our routine practice needs to be well understood and monitored to ensure effectiveness. Indeed, some studies tend to show that AI could increase radiologists’ workload because of a bad understanding or mishandling [37]. Solutions must be found to improve the ergonomic use of AI (such as flagged exams, forcing acknowledgement of AI–human discrepancy), and work must be done in informing and educating radiologists on AI [38].

A limitation of the study is the 137 CT scans excluded due to the lack of available AI reports, which could have caused selection bias. The choice was made to exclude those patients instead of rerunning the AI system, because logistics issues are part of our exercise and should be considered a possibility in any future prospective design and real-world environment. It also enhances the progress to be made in bridging the gap between the efficacy and effectiveness of AI tools.

The main limitation of this study is the low number of positive ICH cases in the night shift subset. It would be interesting to conduct a larger study to support these results. Prospective studies are also required to assess the effectiveness of the AI tool and to measure the impact AI can have on radiologists’ accuracy and clinical improvement in real-world settings. 

## 5. Conclusions

This study shows the great performance of AI intracranial hemorrhage detection and demonstrates even better performance when AI and junior radiologists are combined rather than each one working alone. These results are encouraging for rethinking the radiological workflow and the future of triage of this large population of brain traumatized patients in emergency units. 

## Figures and Tables

**Table 1:** Demographics and clinical characteristics.

Characteristics (n = 682)		
Age	Mean Range; median (IQR)	69.48 18–101; 76 (56–87)
Gender (%)	Female Male	262 (38.4%) 420 (61.6%)
Trauma (%)	Minor Major	570 (83.6%) 112 (16.4%)
Hours (%)	Day shift Night shift	518 (76%) 164 (24%)
By trauma severity		
Major trauma (n = 112; 16.5%)		
Age	Mean Range; median (IQR)	42.34 18–86; 38 (26.75–54)
Gender	Female Male	23 89
Hours	Day shift Night shift	85 27
Minor trauma (n = 570; 83.5%)		
Age	Mean Range; median (IQR)	72.82 18–101; 81 (68–89)
Gender	Female Male	239 331
Hours	Day shift Night shift	433 137
By reading session		
Day shift (n = 518; 76%)		
Age	Mean Range; median (IQR)	70.2 18–101; 76 (59–87)
Gender	Female Male	203 315
Severity	Minor Major	433 85
Night shift (n = 164; 24%)		
Age	Mean Range; median (IQR)	67.1 18–101; 77 (43.75–88)
Gender	Female Male	59 105
Severity	Minor Major	137 27

**Table 2:** Prevalence of intracranial hemorrhage.

	ICH Positive	ICH Negative
Overall (n = 682)	98 (14.4%)	584
By trauma severity		
Major (n = 112) Minor (n = 570)	43 (38.4%) 55 (9.6%)	69 515
By reading session		
Day (n = 518)	77 (14.9%)	441
Major (n = 85) Minor (n = 433)	32 (37.6%) 45 (10.4%)	53 388
Night (n = 164)	21 (12.8%)	143
Major (n = 27) Minor (n = 137)	11 (40.7%) 10 (7.3%)	16 127

**Table 3:** AI performance (IC95%).

**Performance**	**SN**	**SP**	**PPV**	**NPV**	**Accuracy**	**F1 Score**
Overall	88.8% (81–93.6)	92.1% (89.7–94)	65.4% (57–73)	98% (96.5–98.9)	91.6%	90.2%
Minor trauma	85.5% (73.8–92.4)	92.8% (90.3–94.7)	56% (45.3–66.1)	98.4% (96.8–99.2)	92.1%	88.7%
Major trauma	93% (81.4–97.6)	87% (77–93)	81.6% (68.6–90)	95.2% (86.9–98.4)	89.3%	91.1%
**Contingency**	**TP**	**FP**	**TN**	**FN**		
Overall	87	46	538	11		
Minor trauma	47	37	478	8		
Major trauma	40	9	60	3		

AI: artificial intelligence; SN: sensitivity; SP: specificity; PPV: positive predictive value; NPV: negative predictive value; TP: true positive; FP: false positive; TN: true negative; FN: false negative.

**Table 4:** Night shift subset performance (IC95%).

**Performance**	**SN**	**SP**	**PPV**	**NPV**	**Accuracy**	**F1 Score**
AI	90.5% (71.1–97.4)	95.8% (91.2–98.1)	76% (56.6–88.6)	98.6% (94.9–99.6)	95.2%	92.8%
Junior	85.7% (65.4–95)	99.3% (96.2–99.9)	94.7% (75.4–99.1)	98% (94.1–99.3)	97.6%	91.3%
Junior + AI	95.2% (77.3–99.2)	99.3% (96.2–99.9)	95.2% (77.3–99.2)	99.3% (96.2–99.9)	98.8%	97%
**Contingency**	**TP**	**FP**	**TN**	**FN**		
AI	19	6	137	2		
Junior	18	1	142	3		
Junior + AI	20	1	142	1		

AI: artificial intelligence; SN: sensitivity; SP: specificity; PPV: positive predictive value; NPV: nega-tive predictive value; TP: true positive; FP: false positive; TN: true negative; FN: false negative.

**Table 5:** AI false negative clinical details.

Age	Gender	Type	NIRIS Score	GCS	Hospitalization (Days)	Surgery	Outcome
95	F	SAH	1	15	20	No	Death
93	F	SDH	1	15	24	No	LTCF
90	M	SDH	1	15	11	No	LTCF
99	F	IPH	1	15	11	No	Home
60	M	SAH	1	15	2	No	Home
44	M	SDH	1	15	1	No	Home
101	M	SAH	1	15	11	No	Death
83	M	IVH	2	15	4	No	Home
48	M	SAH	1	14	7	No	Home
75	M	SAH + IPH	1	15	7	No	Home
83	M	IPH	1	10	7	No	Home

SAH: subarachnoid hemorrhage; SDH: subdural hemorrhage; IPH: intraparenchymal hemorrhage; IVH: intraventricular hemorrhage; GCS: Glasgow Coma Scale; LTCF: long-term care facility.

## Data Availability

Data is unavailable due to privacy restrictions.

## Supplementary Materials

The following supporting information can be downloaded at: https://www.mdpi.com/article/10.3390/jcm14134403/s1, Data S1: List of radiologists (years of experience); Data S2: AI prefilled report; Figure S1: AI segmentation of anomalies, including ICH, fracture, and midline shift; Figure S2: AI false negative NIRIS 2 case, with a minimal intraventricular hemorrhage; Figure S3: Positive ICH missed by the junior residents, correctly identified by AI.

## Abbreviations

The following abbreviations are used in this manuscript:

ICH	Intracranial hemorrhage
AIH	Acute intracranial hemorrhage
CT	Computed tomography
TBI	Traumatic brain injury
AI	Artificial intelligence
