# Supplementary material for: Real-Life Performance of a Commercially Available AI Tool for Post-Traumatic Intracranial Hemorrhage Detection on CT Scans: A Supportive Tool
Source: J Clin Med. 2025 Jun 20;14(13):4403. doi: 10.3390/jcm14134403 (PMC12249848; doi:10.3390/jcm14134403)

Supplementary Material

Data S1: List of the Radiologist (years of experience)

28 senior radiologists: RG (35), JPT (35), SB (18), VB (20), PC (15), MC (8) , POD (17), NE (20), GF (15), AG (12), CF (11), JCF (41), GH (14), CM (13), MML (35), NR (11), SV (20), JV (15), PB (9), VD (9), TS (7), PQC (8), IMR (7), LV (7), AC (7), MS (6), MV (6), KM (5).
17 junior radiologist residents: MC (5), CT (5), NL (4), JS (4), LM (4), CL (4), JB (4), MP (4), AS (3), YAA (3), FC (2), QA (2), CK (2), PV (2), JLP (2), AB (2), LC (2).

Data S2: AI prefilled report

qER-NCCT - Analyse automatique de TDM crânien

**Nom Patient**	
**ID Patient**	
**Date Scan**	25 Aug 2023
**Date Analyse**	25 Aug 2023 23:10:57
**version qER-NCCT**	1.0.16

Résultat

- **Hémorragie intracérébrale de 13.1 ml**
- **Hémorragie intraparenchymateuse**
- **Hémorragie extradurale**
- **Hémorragie sous-arachnoïdienne**
- **Effet de masse**
- **Décalage de la ligne médiane de 2.5 mm vers la droite**
- **Fracture crânienne**

Coupes Représentatives

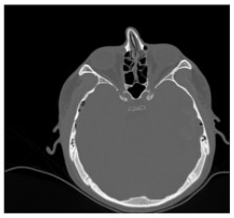

Coupe #70: Hémorragie intracérébrale,Fracture crânienne

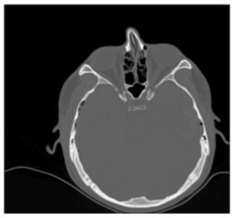

Coupe #71: Hémorragie intracérébrale,Fracture crânienne

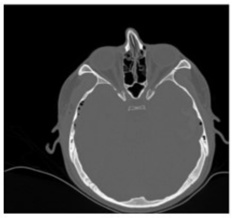

Coupe #72: Hémorragie intracérébrale,Fracture crânienne

Observations

**Anomalies critiques**	**Présence**
**Effet de masse**	Yes
**Décalage de la ligne médiane** de 2.5 mm vers la droite	Yes
**Hémorragie intracérébrale** de 13.1 ml	Yes
**Hémorragie extradurale**	Yes
**Hémorragie intraparenchymateuse**	Yes
Hémorragie intraventriculaire	No
**Hémorragie sous-arachnoïdienne**	Yes
Hémorragie sous-durale	No
**Fracture crânienne**	Yes

Attention: Ceci est un rapport préliminaire généré automatiquement. Il n'est pas destiné à être utilisé seul. Seul le rapport radiologique fait foi.

qure.ai

Figure S1: AI segmentation of anomalies, including ICH, fracture and midline shift

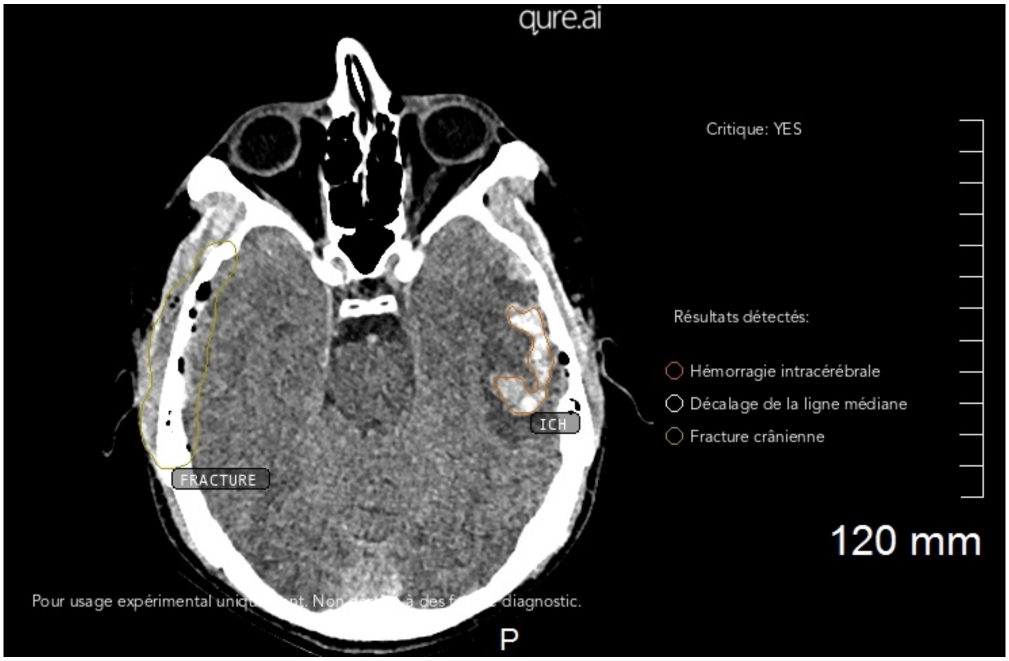

Figure S2: AI false negative NIRIS 2 case, with a minimal intraventricular hemorrhage

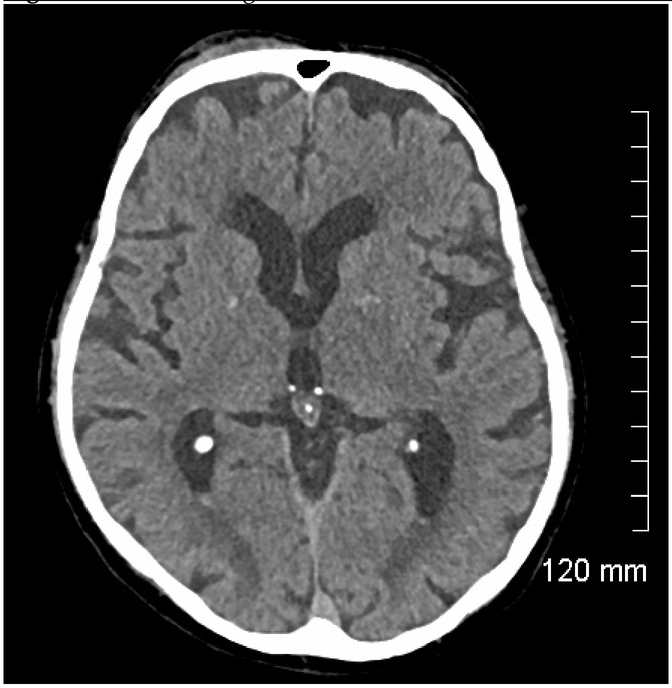

Figure S3: Positive ICH missed by the junior resident, correctly identified by AI

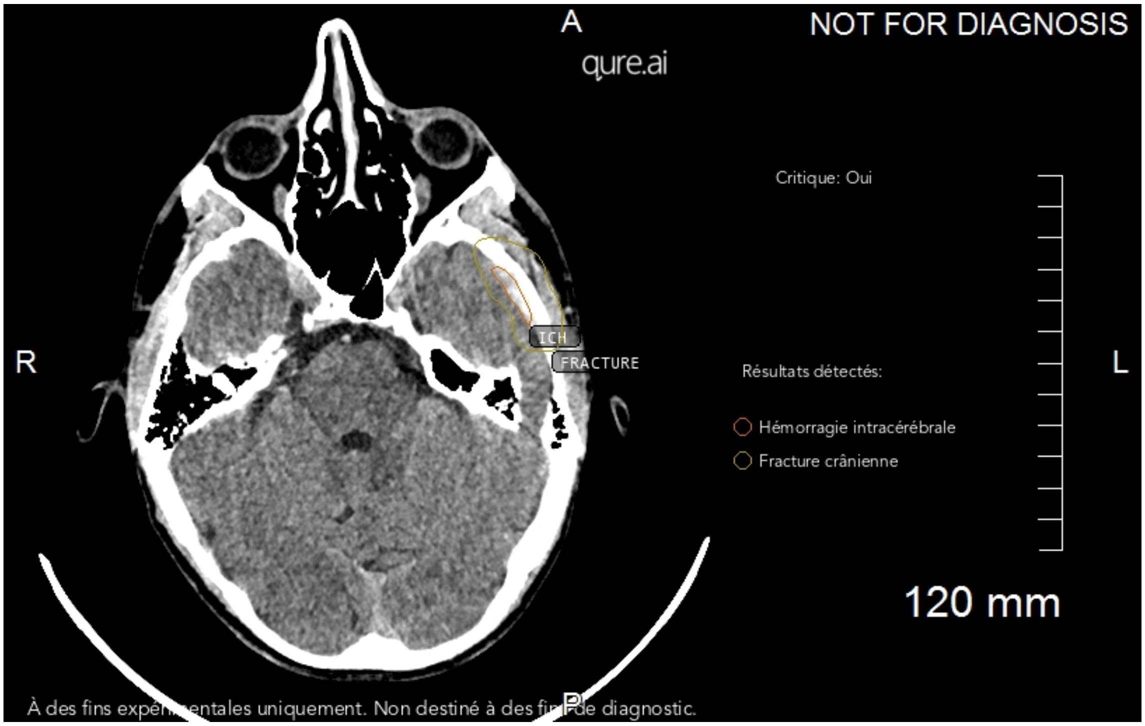

Supplement: Supplementary file 1 [file jcm-14-04403-s001.zip › jcm-3684711-supplementary.pdf]
